# Comparative Diagnostic Performance of a Novel Reverse Transcription Loop-Mediated Isothermal Amplification (RT-LAMP) Kit for the Rapid Detection of SARS-CoV-2

**DOI:** 10.3390/pathogens10121629

**Published:** 2021-12-15

**Authors:** Alexander Domnich, Andrea Orsi, Donatella Panatto, Vanessa De Pace, Valentina Ricucci, Patrizia Caligiuri, Giulia Guarona, Valerio Chessa, Diego Ferone, Simona Boccotti, Bianca Bruzzone, Giancarlo Icardi

**Affiliations:** 1 Hygiene Unit, San Martino Policlinico Hospital-IRCCS for Oncology and Neurosciences, 16132 Genoa, Italy; andrea.orsi@unige.it (A.O.); vanessa.depace@hsanmartino.it (V.D.P.); valentina.ricucci@hsanmartino.it (V.R.); patrizia.caligiuri@hsanmartino.it (P.C.); giuly.guarons@outlook.it (G.G.); valerio.chessa@hsanmartino.it (V.C.); simona.boccotti@edu.unige.it (S.B.); bianca.bruzzone@hsanmartino.it (B.B.); icardi@unige.it (G.I.); 2 Department of Health Sciences, University of Genoa, 16132 Genoa, Italy; panatto@unige.it; 3 Department of Internal Medicine and Medical Specialties, University of Genoa, 16132 Genoa, Italy; ferone@unige.it; 4 Endocrinology Unit, San Martino Policlinico Hospital-IRCCS for Oncology and Neurosciences, 16132 Genoa, Italy

**Keywords:** COVID-19, SARS-CoV-2, RT-LAMP, RT-PCR, diagnostic accuracy

## Abstract

Although the reverse transcription-polymerase chain reaction (RT-PCR) is considered a standard-of-care assay for the laboratory diagnosis of SARS-CoV-2, several limitations of this method have been described. Reverse transcription loop-mediated isothermal amplification (RT-LAMP) is an alternative molecular assay and is potentially able to overcome some intrinsic shortcomings of RT-PCR. In this study, we evaluated the diagnostic performance of the novel HG COVID-19 RT-LAMP assay. In this retrospective analysis, a total of 400 routinely collected leftover nasopharyngeal samples with a known RT-PCR result were tested by means of the HG COVID-19 RT-LAMP assay. The overall sensitivity and specificity values of HG COVID-19 RT-LAMP versus RT-PCR were 97.0% (95% CI: 93.6–98.9%) and 98.5% (95% CI: 95.7–99.7%), respectively. Inter-assay agreement was almost perfect (*κ* = 0.96). Concordance was perfect in samples with high viral loads (cycle threshold < 30). The average time to a positive result on RT-LAMP was 17 min. HG COVID-19 RT-LAMP is a reliable molecular diagnostic kit for detecting SARS-CoV-2, and its performance is comparable to that of RT-PCR. Shorter turnaround times and the possibility of performing molecular diagnostics in the point-of-care setting make it a valuable option for facilities without sophisticated laboratory equipment.

## 1. Introduction

The precise laboratory diagnosis of SARS-CoV-2 is crucial to tackling the ongoing COVID-19 pandemic. The laboratory diagnosis of both symptomatic and asymptomatic SARS-CoV-2 cases can be achieved by means of both direct (detection of the viral RNA or antigens) and indirect (detection of specific antibodies) approaches [1,2,3].

Reverse transcription-polymerase chain reaction (RT-PCR) is considered the standard method for the laboratory diagnosis of SARS-CoV-2 infection, and several protocols for its execution have been developed [1,4,5]. However, while the specificity of RT-PCR is deemed high [6], some issues regarding its sensitivity have been reported. Indeed, a systematic review by Arevalo-Rodriguez et al. [7] has shown that the false negativity rate (defined as initial negative result followed by a positive result) may vary from 1.8% to 58% (median of 11%). Other potential shortcomings of the RT-PCR assay include the need for both qualified personnel and sophisticated laboratory equipment, its still suboptimal turnaround time and its relatively high per-sample costs [3].

In order to overcome the abovementioned intrinsic limits of RT-PCR, some alternative direct methods have been promptly developed [1,2,3]. Among these, antigen-detecting rapid diagnostic tests (Ag-RDTs) have become popular, and dozens of kits have been commercialized. However, as shown by a recent Cochrane review [8], the diagnostic performance of Ag-RDTs is highly variable and depends on several factors, including, for example, viral load, presence or absence of COVID-19 symptoms, time since the onset of symptoms and brand.

The loop-mediated isothermal amplification (LAMP) assay is another alternative to RT-PCR for the molecular diagnosis of SARS-CoV-2. LAMP of DNA was originally described in 2000 [9], and since that time, this method has gained prominence as a rapid, accurate and cost-effective diagnostic method for a variety of pathogens [10,11]. Typical LAMP reagents comprise salts, nucleotides, DNA polymerase and a set of 4–6 primers, including loop primers, forward and reverse inner primers and outer primers [12]. Reverse transcription LAMP (RT-LAMP) protocols have been developed to detect RNA virus sequences by adding a heat-stable reverse transcriptase enzyme to the LAMP mixture [13]. Compared with RT-PCR, the LAMP technique can rapidly amplify and produce up to 100 times more nucleic acid copies in isothermal conditions of 60–65 °C [12].

The first available data suggest that RT-LAMP assays have a high diagnostic performance in detecting SARS-CoV-2. In particular, a recent systematic review and meta-analysis [14] showed pooled sensitivity values of RT-LAMP versus RT-PCR of 94% (95% CI: 90–96%) and 78% (95% CI: 65–87%) for extracted and fresh samples, respectively. Specificity was 100% (95% CI: 99–100%) and 96% (95% CI: 95–99%) for extracted and fresh specimens, respectively.

The HG COVID-19 assay (HiberGene Diagnostics, Republic of Ireland) is among the first RT-LAMP assays intended for the rapid molecular diagnosis of SARS-CoV-2. The development and commercialization of this assay were supported by the Horizon 2020 Research and Innovation program [15]. The objective of the present study was to assess the relative (compared with RT-PCR) diagnostic performance of the HG COVID-19 assay in routinely processed nasopharyngeal (NP) specimens.

## 2. Results

A total of 400 (200 positive and 200 negative) RT-PCR NP swabs were analyzed in the present study (Supplementary Materials Figure S1). The mean age of subjects was 50.4 ± 21.8 years; 52.5% (95% CI: 47.5–57.5%) were females. RT-PCR-positive and -negative subjects were similar in terms of age (49.7 ± 21.8 vs. 51.0 ± 21.8 years, respectively; *p* = 0.56] and sex [50.0% (95% CI: 42.9–57.1%) vs. 55.0% (95% CI: 47.8–62.0%) of females, respectively; *p* = 0.37]. Among RT-PCR-positive samples, Ct values for the N gene ranged from 15 to 39, with an average of 24.9 ± 5.7. Among the samples tested for lineages (*n* = 64), all but one [98.4% (95% CI: 91.6–100%)] belonged to the Alpha variant of concern.

Table 1 reports the raw data on the comparison between the RT-PCR and HG COVID-19 RT-LAMP assays in terms of SARS-CoV-2 detection in NP samples. Briefly, a total of nine [2.3% (95% CI: 1.0–4.2%)] discordant results were documented: six [3.0% (95% CI: 1.1–6.4%)] were judged false negatives, while the other three [1.5% (95% CI: 0.3–4.3%)] were false positives. Therefore, the overall diagnostic accuracy, sensitivity and specificity values were 97.8%, 97.0% and 98.5%, respectively. Moreover, the overall inter-assay concordance was judged almost perfect (*κ* = 0.96) (Table 2).

The nine discordant results were then analyzed in detail. All six NP swabs that tested negative on RT-PCR had N gene cycle threshold (Ct) values > 30 (range: 31–38). Indeed, as shown in Table 3, the relative sensitivity of the HG COVID-19 assay was 100% for NP samples with Ct < 30 and 97.0% for those ≤ 39. By contrast, of the three false-positive NP samples, two had tested positive on RT-PCR 1–3 weeks earlier with Ct values > 30. The sensitivity for low viral load samples (Ct ≥ 30) was 88.0% (95% CI: 76.2–94.4%).

**Figure 1:**
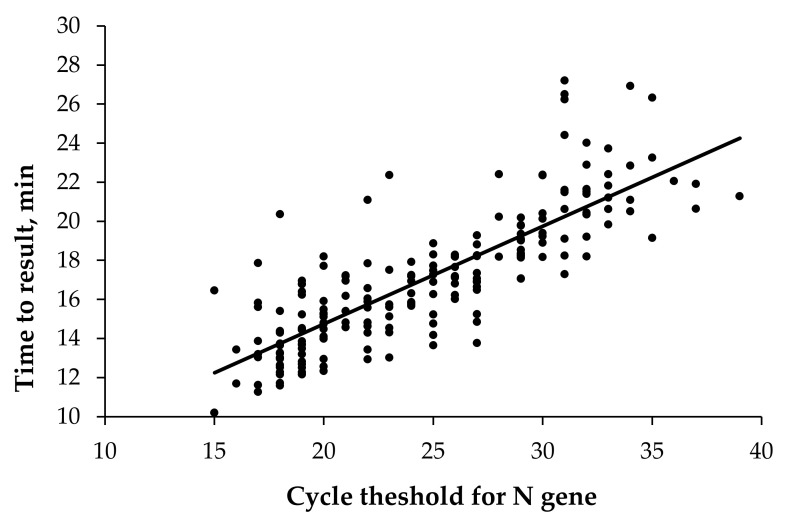
Correlation between N gene cycle threshold values and time to positive result on the HG COVID-19 RT-LAMP assay.

Among the true positive samples (*n* = 194), the average time to result on the HG COVID-19 RT-LAMP assay was 17.0 ± 3.4 min. As expected, there was a clear (*r* = 0.82, *p* < 0.001) relationship between the nucleoprotein (N) gene Ct value and the time to the result of the HG COVID-19 assay (Figure 1). Indeed, the average time to result for samples with high viral loads (Ct < 20) was only 13.9 ± 1.9 min.

## 3. Discussion

This study is among the first independent on-field evaluations of the HG COVID-19 RT-LAMP assays. Our findings confirmed the high diagnostic performance of this assay in detecting SARS-CoV-2 in NP samples with a wide (15–39) range of Ct values. We also demonstrated that the recent predominance of the Alpha variant of concern did not affect the analytical performance of the kit. Indeed, the HG COVID-19 assay targets the highly conserved N region, while the novel variants of concern present key mutations in the S region. These mutations may affect the diagnostic performance of RT-PCR; indeed, so-called “S-gene target failure” is very common in samples positive for the Alpha variant of concern [16,17]. Finally, our study is the first to validate the HG COVID-19 assay on an alternative extraction platform, which shows that RT-LAMP can be run successfully on different types of laboratory equipment.

According to the manufacturer’s instructions for use, the relative (vs. RT-PCR) sensitivity and specificity values of the HG COVID-19 extracted format in symptomatic patients are 100% (95% CI: 84.5–100%) and 100% (95% CI: 89.1–100%), respectively. In asymptomatic individuals, these parameters are 88.5% (95% CI: 75.9–95.2%) and 97.1% (95% CI: 92.9–98.9%), respectively. Considering that our sample included both symptomatic and asymptomatic subjects, our findings are consistent with the on-label analytical performance and testify to the generalizability of our results. On the other hand, for low viral load samples (Ct ≥ 30), the relative sensitivity RT-LAMP vs. RT-PCR dropped to 88%.

Our results are also in line with the meta-analytical estimates obtained by Subsoontorn et al. [14]. In their systematic review, the pooled (*n* = 26 studies) sensitivity and specificity values of the extracted RT-LAMP format versus RT-PCR were 94% (95% CI: 90–96%) and 100% (95% CI: 99–100%), respectively. When the analysis was restricted to high viral load samples (Ct < 30), the pooled estimates (*n* = 10 studies) were 100% (95% CI: 89–100%) and 100% (95% CI: 99–100%), respectively [14]. In our study, while some false negatives with high RT-PCR Ct values (>30) were expected, some comments on the “false positives” should be made. It has been suggested that some non-specific amplifications may occur during RT-LAMP [18]. We documented a total of three false-positive results; of these, however, two subjects had proved positive on RT-PCR 1–3 weeks earlier. On the one hand, it is well-known that the RT-LAMP technique can amplify up to 100 times more RNA copies than RT-PCR [12]. On the other hand, the RT-PCR assay used in this study (Allplex 2019-nCoV; Seegene Inc., Seoul, South Korea) considers samples with Ct ≤ 40 to be positive [19]. It is therefore likely that the two abovementioned subjects with false-positive results could have had Ct values over 40. In turn, this may also mean that the reported specificity of the HG COVID-19 assay might have been underestimated. There is an ongoing debate on the association between infectiousness and RT-PCR Ct values. It is generally believed that infectiousness is significantly lower for clinical specimens with Ct values > 30 [20,21]. However, a substantial number of samples with Ct > 35 may still produce a viable virus [22,23,24]. Moreover, Ct values across different RT-PCR protocols may vary significantly [25].

The possibility of rapidly obtaining a precise result makes RT-LAMP technology an attractive point-of-care or near-the-patient tool. This feature of RT-LAMP resembles that of Ag-RDTs. The World Health Organization [26] recommends that SARS-CoV-2 Ag-RDTs should have sensitivity and specificity values of at least 80% and 97%, respectively, while the European Centre for Disease Control and Prevention [20] has suggested that the sensitivity of point-of-care tests should be at least 90%. The HG COVID assay satisfies these criteria. An assessment of several Ag-RDTs recently performed at our laboratory [27] showed overall sensitivity of 78.7% (95% CI: 73.2–83.3%); this, however, varied significantly by Ct value and brand. We concluded that Ag-RDTs are convenient for screening purposes in moderate-to-high intensity settings [27]. In the present study, the analytical performance of the HG COVID-19 assay was comparable to that of RT-PCR; the assay may therefore be an alternative molecular diagnostic tool for SARS-CoV-2 detection, independently of the purpose and the viral epidemiology. On the other hand, it is likely that not all commercially available RT-LAMP kits perform well. For instance, a real-world evaluation of the diagnostic performance of the Isopollo COVID-19 RT-LAMP assay (M Monitor, Daegu, Korea) showed a sensitivity of only 61.9% [28]. Independent and setting-specific pilot evaluations would therefore be beneficial before the widespread implementation of RT-LAMP assays.

Like that of most available Ag-RDTs, the HG COVID-19 readout is qualitative. The absence of Ct values in the final readout may be seen as an intrinsic limitation of this method. However, the time to threshold is a good proxy of viral load. Indeed, we observed a strong linear association between the HG COVID-19 time to result and RT-PCR Ct values. Results that are available in less than 15–20 min are highly suggestive of high viral loads (Ct < 30). Although no RT-PCR samples with inconclusive results were tested, the short turnaround time of the RT-LAMP assay makes it attractive for use as a “resolver” test.

Our study is not without limitations. First, for ethical reasons, we were not able to link the RT-PCR readout to the clinical characteristics of patients (e.g., presence of symptoms, days after the onset of symptoms, etc.). According to the manufacturer’s instructions for use, the performance of the HG-COVID assay is better in symptomatic cases, especially when the test is performed soon after the onset of symptoms. Second, we cannot completely rule out misclassification bias. Although RT-PCR is currently considered the “gold standard” assay for the laboratory diagnosis of both symptomatic and asymptomatic cases [1,4,5], its sensitivity is not perfect [7]. In our study, two samples from patients with previously confirmed SARS-CoV-2 infection were positive on RT-LAMP but negative on RT-PCR. This means that, in some instances, RT-LAMP may be more sensitive than RT-PCR, and that the reported relative specificity of RT-LAMP might have been underestimated. Third, the study was carried out in a period when the Delta variant of concern did not circulate in Italy. We, however, believe that this possible limitation has a limited impact on the study conclusions for two reasons. First, the HG COVID-19 assay targets the highly conserved N region, while the key mutations of the Delta variant are located in the S region. Second, our subsequent routine use of the HG COVID-19 assay was able to detect isolates belonging to the Delta variant (results not shown). Another possible shortcoming is that RT-PCR Ct values may differ by brand and protocol; the reported diagnostic accuracy estimates by the Ct category may differ from those observed in other laboratories. For instance, we have previously shown [25] that compared with the extraction-based RT-PCR technique, the extraction-free protocol adopted in the present study is associated with an average increase in Ct values by 2–3 units. Finally, although highly conserved, the N gene may be subject to mutations affecting molecular diagnostics of SARS-CoV-2 infection [29]. Future studies should explore the diagnostic accuracy of the HG COVID-19 assay for isolates presenting, for example, the N gene target failure on the commercially available RT-PCR kits.

In conclusion, HG COVID-19 RT-LAMP is a reliable assay for the molecular diagnosis of SARS-CoV-2 in NP samples and yields a final diagnosis in less time than RT-PCR. RT-LAMP technology is promising for use in small and medium-sized hospitals, emergency departments and general practices without sophisticated laboratory equipment.

## 4. Materials and Methods

### 4.1. Reporting Quality

For the purpose of reporting, we adopted the STARD (Standards for Reporting of Diagnostic Accuracy Studies) statement [30]. The checklist is available in Supplementary Materials Table S1.

### 4.2. Overall Study Design, Sampling and Setting

The NP samples analyzed in this study came from routine SARS-CoV-2 molecular diagnostics and were collected between 8 April and 9 May 2021. All samples were processed at the regional reference laboratory for COVID-19 diagnostics, located at San Martino Policlinico Hospital, Hygiene Unit (Genoa, Italy). This laboratory performs SARS-CoV-2 RT-PCR on both in- and outpatient specimens, independently of the purpose of testing (laboratory confirmation of symptomatic cases, screening, clinical follow-up, etc.). No clinical data associated with a given sample were available. All samples were taken by means of a flocked swab kit and were eluted in universal transport medium (UTM, Copan Diagnostics Inc., Murrieta, CA, USA).

The index test was the HG COVID-19 assay, while the reference test was RT-PCR, which is currently considered the “gold standard” technique for the laboratory diagnosis of SARS-CoV-2 [1,4,5]. Following a successful RT-PCR run, the anonymized leftover samples were tested by means of RT-LAMP, and therefore the positivity status was known beforehand. All RT-PCR tests were executed within 8 h of the arrival of specimens at the laboratory.

Any NP samples with a known RT-PCR readout were potentially eligible for the study. Specimens showing Ct values ≤ 40 were deemed positive. This cut-off was chosen on the basis of the RT-PCR assay available at our laboratory and considers samples with Ct ≤ 40 to be positive [19].

The sample size of 400 (200 positive and 200 negative RT-PCR samples) specimens was judged sufficiently powered to assess the diagnostic performance of the HG COVID-19 assay. Indeed, according to the Foundation for Innovative New Diagnostics [31], a minimum of 100 RT-PCR positive and 100 RT-PCR negative samples is needed for the comparative evaluation of Ag-RDTs. Two-hundred routinely analyzed positive specimens were collected by means of quota sampling. Specifically, on the basis of RT-PCR Ct values, positive samples were first categorized into four groups: (i) <20; (ii) 20–24.9; (iii) 25–29.9 and (iv) ≥ 30, and consecutively fulfilled in a 1:1:1:1 ratio (i.e., 50 specimens per group). Given that the HD COVID RT-LAMP assay targets the N gene, samples were categorized according to the RT-PCR Ct value of this gene. All 200 negative samples were gathered according to convenience.

During the study period, most (>90%) detections (as shown by a random sample of specimens sequenced by our laboratory on a regular basis) were attributable to the Alpha variant of concern of SARS-CoV-2. For the purpose of this study, we further analyzed a sample of 64 RT-PCR-positive specimens (Ct < 30) to determine their lineage. This was done by means of the RT-PCR-based Variant Catcher system by Clonit (Milan, Italy), which is able to distinguish between the so-called wild-type (Wuhan-like) and Alpha and Beta/Gamma variants of concern.

### 4.3. Real-Time Reverse Transcription-Polymerase Chain Reaction (RT-PCR)

As per the internal SARS-CoV-2 diagnostic protocol adopted by the San Martino Policlinico Hospital, all NP samples were processed by means of a validated [25] unheated RNA extraction-free RT-PCR method. We have previously shown [25] that this technique displays perfect agreement with a traditional extraction-based approach. Briefly, an input volume of 5 µl of specimen was first diluted (1:3) and set up for RT-PCR. RT-PCR was run on a CFX96 instrument (Bio-Rad Laboratories, Hercules, CA, US) by using the Allplex 2019-nCoV (Seegene Inc., Seoul, South Korea) assay. This multiplex assay is able to simultaneously detect three gene targets, namely N, RNA-dependent RNA-polymerase (RdRp)/spike (S) and envelope (E) regions. Amplification was performed at 50 °C for 20 min, 95 °C for 15 min, 45 cycles at 95 °C for 10 s, 60 °C for 15 s with first acquisition and 72 °C for 10 s with second acquisition on the CFX96 thermal cycler. A total of 5 µL of the extracted RNA in a final volume of 20 µL was used for each reaction. The average time-to-result of the unheated RNA extraction-free RT-PCR method is 156 min [25].

For the purpose of this study, only “valid” (i.e., when the internal control successfully amplified) RT-PCR samples were eligible.

### 4.4. HG COVID-19 Assay

The index test was the HG COVID-19 (HiberGene Diagnostics, Dublin, Ireland) assay. This is a LAMP-based assay that targets the highly conserved N region. The reaction strips contain both complete lyophilized reaction mixes for SARS-CoV-2 and an extraction control, which is used to demonstrate the absence of inhibitors. The HG COVID-19 assay can be performed in two formats: (i) direct (i.e., from the fresh sample) and (ii) extracted. In this validation study, the extracted option was used. Total RNA of NP specimens was extracted by means of the STARMag Universal Cartridge Kit (Seegene Inc., Seoul, Korea) on the automated Nimbus IVD (Seegene Inc., Seoul, Korea) platform. The extracts obtained were eluted in 140 µL of the viral elution buffer provided with the kit.

Subsequently, a total of 25 μL of the lysed samples obtained was added to target and control wells, vortexed for 5 s and centrifuged for 10 s at 3000 rcf. The reaction was run on the HG Swift instrument (HiberGene Diagnostics, Dublin, Ireland). 

The final readout is qualitative and may be displayed as “positive”, “negative” or “invalid”. According to the manufacturer, positive results are available in < 30 min, while negative results can be recorded in < 60 min. For positive samples, the time to result was automatically collected. In the case of an inconclusive result, the test was repeated.

### 4.5. Data Analysis

Categorical variables are expressed as proportions with 95% CIs, and approximately normally distributed continuous variables as means ± standard deviations. Chi-square and *t*-tests were used to compare proportions and continuous variables, respectively. There were no missing data (Supplementary Materials Dataset S1).

The diagnostic performance of the HG COVID-19 assay was compared with the output of RT-PCR in terms of overall diagnostic accuracy, Cohen’s *κ*, sensitivity and specificity. These statistics were also calculated according to the RT-PCR Ct values for the N gene.

For true positive samples, the average time to the result of the HG COVID-19 assay was calculated. Pearson’s *r* coefficient was used to establish a correlation between the RT-PCR Ct value for the N gene and time to the result provided by RT-LAMP.

The raw dataset used is available in Supplementary Materials Dataset S1. All analyses were performed in R stats packages v. 4.0.3 (R Foundation for Statistical Computing, Vienna, Austria) [32].

## Figures and Tables

**Table 1:** Two-per-two table on the performance of the HG COVID-19 RT-LAMP assay, as compared with RT-PCR (Results are reported as % (*n*)).

RT-LAMP	RT-PCR		Total
	Positive	Negative	
**Positive**	97.0 (194)	1.5 (3)	49.3 (197)
**Negative**	3.0 (6)	98.5 (197)	50.7 (203)
**Total**	50.0 (200)	50.0 (200)	100 (400)

**Table 2:** Diagnostic performance indicators of the HG COVID-19 RT-LAMP assay, as compared with RT-PCR.

Parameter	Estimate	95% CI
**Diagnostic accuracy, %**	97.8	95.8–99.0
**Sensitivity, %**	97.0	93.6–98.9
**Specificity, %**	98.5	95.7–99.7
**Cohen’s** ***κ***	0.96	0.86–1

**Table 3:** Sensitivity of the HG COVID-19 RT-LAMP assay, as compared with RT-PCR, by N gene cycle threshold value.

N Gene Cycle Threshold (*n*)	Estimate, %	95% CI
<20 (50)	100	92.3–100
<25 (100)	100	96.4–100
<30 (150)	100	97.6–100
<33 (178)	99.4	96.9–100
<36 (195)	97.4	94.1–99.2
≤39 (200)	97.0	93.6–98.9

## Data Availability

Raw data are available within the manuscript (Supplementary Materials Dataset S1).

## Supplementary Materials

The following are available online at https://www.mdpi.com/article/10.3390/pathogens10121629/s1, Figure S1: STARD (Standards for Reporting of Diagnostic Accuracy Studies) flowchart; Table S1: STARD (Standards for Reporting of Diagnostic Accuracy Studies) checklist; Dataset S1: Raw data used in the study.
