# Supplementary material for: Comparative Diagnostic Performance of a Novel Reverse Transcription Loop-Mediated Isothermal Amplification (RT-LAMP) Kit for the Rapid Detection of SARS-CoV-2
Source: Pathogens. 2021 Dec 15;10(12):1629. doi: 10.3390/pathogens10121629 (PMC8706056; doi:10.3390/pathogens10121629)
Supplement: Supplementary file 1 [file pathogens-10-01629-s001.zip › Table_S1.pdf]

Table S1. STARD (Standards for Reporting of Diagnostic Accuracy Studies) checklist.

Section & Topic	No	Item	Reported in section (§)
**TITLE OR ABSTRACT**	**1**	Identification as a study of diagnostic accuracy using at least one measure of accuracy (such as sensitivity, specificity, predictive values, or AUC)	Title (NA)
**ABSTRACT**	**2**	Structured summary of study design, methods, results, and conclusions (for specific guidance, see STARD for Abstracts)	Abstract (NA)
**INTRODUCTION**			
	**3**	Scientific and clinical background, including the intended use and clinical role of the index test	1 (1–7)
	**4**	Study objectives and hypotheses	1 (7)
**METHODS**			
Study design	**5**	Whether data collection was planned before the index test and reference standard were performed (prospective study) or after (retrospective study)	4.2 (2)
Participants	**6**	Eligibility criteria	4.2 (3)
	**7**	On what basis potentially eligible participants were identified (such as symptoms, results from previous tests, inclusion in registry)	4.2 (3, 4)
	**8**	Where and when potentially eligible participants were identified (setting, location and dates)	4.2 (1)
	**9**	Whether participants formed a consecutive, random or convenience series	4.2 (2, 4)
Test methods	**10a**	Index test, in sufficient detail to allow replication	4.4 (1, 2)
	**10b**	Reference standard, in sufficient detail to allow replication	4.3 (1)
	**11**	Rationale for choosing the reference standard (if alternatives exist)	4.2 (2)
	**12a**	Definition of and rationale for test positivity cut-offs or result categories of the index test, distinguishing pre-specified from exploratory	4.4 (3)
	**12b**	Definition of and rationale for test positivity cut-offs or result categories of the reference standard, distinguishing pre-specified from exploratory	4.3 (2) and 4.2 (4)
	**13a**	Whether clinical information and reference standard results were available to the performers/readers of the index test	4.2 (1, 2)
	**13b**	Whether clinical information and index test results were available to the assessors of the reference standard	4.2 (1, 2)
Analysis	**14**	Methods for estimating or comparing measures of diagnostic accuracy	4.5 (2)
	**15**	How indeterminate index test or reference standard results were handled	4.4 (3) and 4.3 (2)
	**16**	How missing data on the index test and reference standard were handled	4.5 (1)
	**17**	Any analyses of variability in diagnostic accuracy, distinguishing pre-specified from exploratory	4.5 (2)
	**18**	Intended sample size and how it was determined	4.2 (4)
**RESULTS**			
Participants	**19**	Flow of participants, using a diagram	Figure S1
	**20**	Baseline demographic and clinical characteristics of participants	2 (1)
	**21a**	Distribution of severity of disease in those with the target condition	4.2 (4)

	**21b**	Distribution of alternative diagnoses in those without the target condition	Figure S1
	**22**	Time interval and any clinical interventions between index test and reference standard	4.2 (1)
Test results	**23**	Cross tabulation of the index test results (or their distribution) by the results of the reference standard	1 (Table 1)
	**24**	Estimates of diagnostic accuracy and their precision (such as 95% confidence intervals)	1 (Tables 2 and 3)
	**25**	Any adverse events from performing the index test or the reference standard	NA
**DISCUSSION**			
	**26**	Study limitations, including sources of potential bias, statistical uncertainty, and generalisability	3 (6)
	**27**	Implications for practice, including the intended use and clinical role of the index test	3 (1,4,5,)
**OTHER INFORMATION**			
	**28**	Registration number and name of registry	See the note below
	**29**	Where the full study protocol can be accessed	See the note below
	**30**	Sources of funding and other support; role of funders	Funding

Note: The study aim and design is described in the grant agreement No 101003713; NA, not applicable.
