# Supplementary figures and images for: Comparative Diagnostic Performance of a Novel Reverse Transcription Loop-Mediated Isothermal Amplification (RT-LAMP) Kit for the Rapid Detection of SARS-CoV-2
Source: Pathogens. 2021 Dec 15;10(12):1629. doi: 10.3390/pathogens10121629 (PMC8706056; doi:10.3390/pathogens10121629)

**Figure S1.** STARD (Standards for Reporting of Diagnostic Accuracy Studies) flowchart.

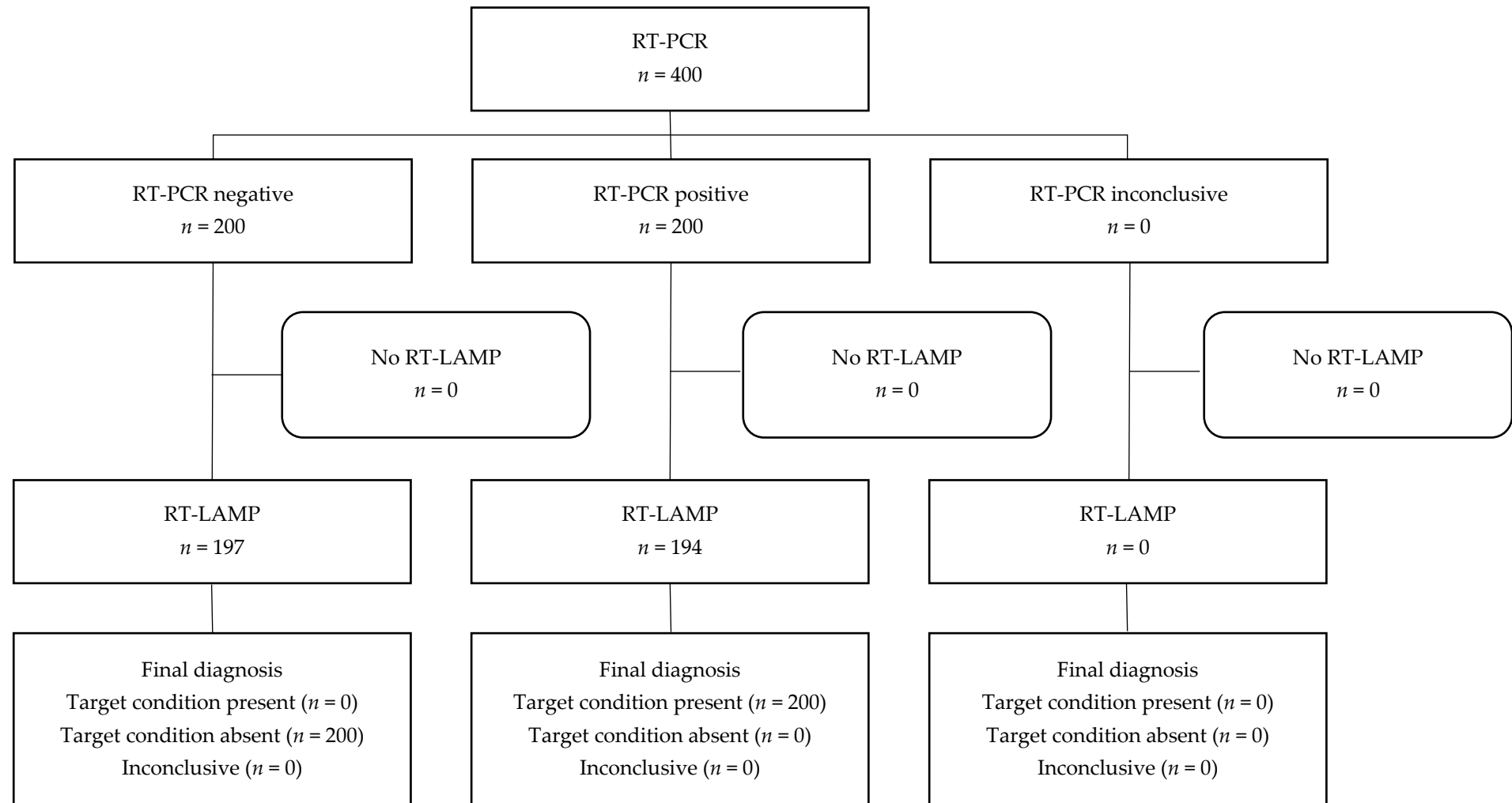

Supplement: Supplementary file 1 [file pathogens-10-01629-s001.zip › Figure_S1.pdf]
